# Outcomes of HeartMate 3 in pediatric patients with end-stage heart failure: a single-center preliminary experience from Turkey

**DOI:** 10.3389/fcvm.2024.1472663

**Published:** 2024-10-14

**Authors:** Eser Doğan, Zulal Ulger Tutar, Osman Nuri Tuncer, Reşit E. Levent, Çağatay Engin, Tahir Yağdı, Yüksel Atay, Mustafa Özbaran

**Affiliations:** ^1^ Department of Pediatric Cardiology, Faculty of Medicine, Ege University, İzmir, Türkiye; ^2^ Department of Cardiovascular Surgery, Faculty of Medicine, Ege University, İzmir, Türkiye

**Keywords:** cardiac transplant, heart failure, HeartMate 3, pediatric, Turkey

## Abstract

**Objectives:**

We aim to evaluate our initial experience with the HeartMate 3 (HM3) device (Abbott, USA) for palliating pediatric patients with end−stage heart failure (ESHF).

**Methods:**

We conducted a retrospective review of clinical data from pediatric patients (aged 7–18 years) who underwent HM3 implantation for ESHF at our institution between 2022 and 2024. Patient demographics and follow−up data were comprehensively analyzed.

**Results:**

We identified 11 patients (45% males) with a median age of 14 years (IQR 11–17), a median weight of 47 kg (IQR 28–50), a median height of 159 cm (IQR 135–165), and a median body surface area of 1.36 m^2^ (IQR 1.07–1.53) at the time of the intervention. All patients were diagnosed with dilated cardiomyopathy and categorized with PEDIMACS profiles ranging from one to three. The median ICU stay was 14 days (IQR 6–32), with 11 patients receiving inotropic support for a median of four postoperative days (IQR 3–8). The median follow−up period was 150 days (IQR 90–210). Early complications included two cases of pleural effusion, 1 case of cardiac tamponade, 3 cases of polyuria, and one instance of positive blood cultures. One patient, who was non−compliant with warfarin therapy, developed a thrombus in the right atrium that was resolved with a revision of anticoagulant therapy, and did not experience pump thrombosis. During follow−up, one patient died after 28 days from sepsis, one underwent heart transplantation after 10 days, and nine patients remained alive on the device. Notably, there were no reported cases of pump thrombosis, ischemia, or stroke post− implantation.

**Conclusions:**

The HM3 device appears to be a safe and effective palliative option for pediatric patients with ESHF.

## Introduction

Pediatric heart failure is a significant source of morbidity and mortality during childhood. For patients with end−stage heart failure (ESHF), heart transplantation is the optimal treatment option. However, ventricular assist devices (VADs) are utilized as a bridge to transplantation (due to difficulties in finding organ donors), for myocardial recovery (in cases of myocarditis), or as a destination therapy (in patients with systemic diseases) (1). In recent years, VADs have not only served as a bridge to transplantation for patients with ESHF but have also aided in cardiac recovery and even become a target therapy option for long−term treatment (2). Additionally, VADs aim to sustain life until a transplantation opportunity arises and mitigate the detrimental effects of heart failure on organs and systems. Although VADs are known to enhance survival, functional capacity, and quality of life in ESHF, they also pose significant morbidity risks due to repeated hospital admissions, infections, bleeding, and thrombosis (3, 4). Bleeding, pump thrombosis, stroke, right ventricular failure, and hemolysis are factors that limit VAD therapy. Improving the hemocompatibility of VADs is essential for reducing morbidity and mortality. The HeartMate 3 (HM3) device (Abbott Corp, USA) is a novel VAD that aims to overcome these complications with its fully magnetically levitated, continuous centrifugal flow design (5, 6). However, the mismatch between device size and patient size limits the use of HM3 in the pediatric patient population, resulting in a limited number of studies reporting experiences with HM3 in children (7). We aim to present our preliminary experience with HM3 implantation in pediatric patients with ESHF.

## Methods

### Study design and patient selection

We conducted a retrospective clinical data review of all pediatric patients (aged 7–18 years) who underwent HM3 implantation for ESHF in our institution, between January 2022 and March 2024. Clinical, procedural, and follow−up data were collected and comprehensively analyzed. Ethical approval was obtained from the institutional review board. Written informed consent was obtained from patients or their legal guardians for the procedure and the use of clinical records for publication purposes.

### Diagnosis of end−stage heart failure

Patients were evaluated by a multidisciplinary team and underwent comprehensive investigations to determine underlying etiologies prior to being scheduled for HM3 implantation. Patients were also assessed according to PEDIMACS profiles (8). Although there are clear guidelines for heart transplant listing, universally accepted criteria for HM3 implantation do not exist. We implanted the HM3 in patients meeting the following criteria: (1) presence of New York Heart Association (NYHA) class IIIb−IV symptoms for at least 45 out of the last 60 days, (2) heart failure symptoms unresponsive to optimal medical therapy, (3) left ventricular ejection fraction <25%, (4) peak oxygen consumption <14 ml/kg/min or a continued need for intravenous (IV) inotropic therapy due to symptomatic hypotension, declining renal function, or worsening pulmonary congestion, (5) IV inotropic drug use for ≥14 days, and (6) intra−aortic balloon pump support for ≥7 days.

### HeartMate 3 device

**Figure 1:**
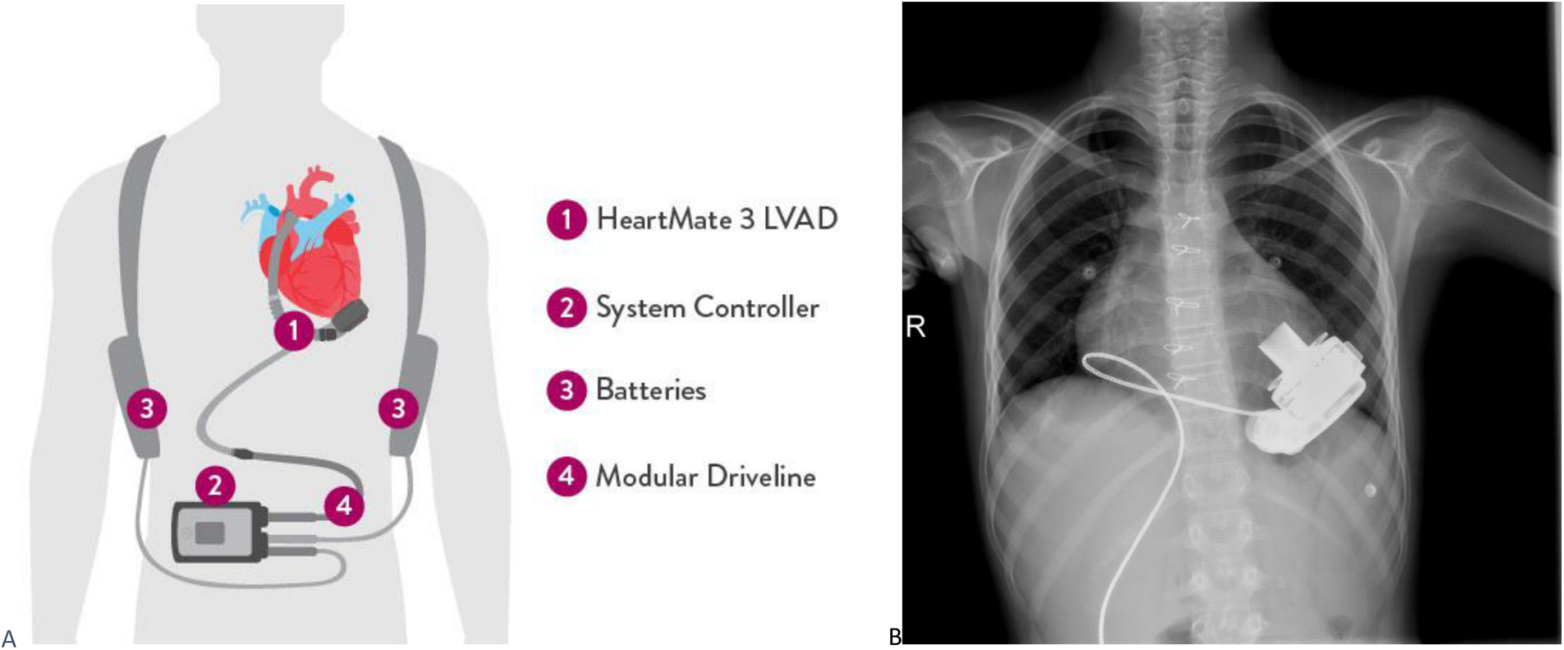
**(A)** Heartmate 3 device and equipments are shown. **(B)** PA chest radiograph of a pediatric patient implanted with HM3.

The HM3 (Figure 1) is a fully magnetically levitated LVAD and has 4 unique features: (1) a fully magnetically levitated rotor, (2) large blood flow pathways, (3) intrinsic pulsatility, and (4) an intradevice operating system. The rotor is fully levitated and self-centered, without the need for hydrodynamic or mechanical bearings. This Full MagLev technology (Abbott Corp, USA) decreases the shear stress and compressive forces seen with hydrodynamic bearings (9). Additionally, the HM3 has large, consistent blood flow pathways owing to the rotor and inlet design. Although hydrodynamic bearing rotors and inlets have narrow blood flow pathways, the HM3 inlet pathways are 10 20 times larger. These larger pathways minimize shear stress, avoid stasis, and decrease activation of thrombogenic blood components (9).

### HM3 device implantation protocol

Implantations were performed through a median sternotomy, with cardiopulmonary bypass, through cannulation of ascending aorta and right atrium on beating heart without cardioplegic arrest. Inflow cannulation was inserted through the left ventricular apex. The outflow cannula was sutured to the proximal ascending aorta with the assistance of a side−biting clamp. Postoperatively, all patients were monitored in the cardiovascular surgery intensive care unit (ICU) for the first 48 h before being transferred to the Pediatric Intensive Care Unit. Patients received intravenous inotropic support including adrenaline, noradrenaline, dopamine, milrinone, and dobutamine, which were gradually tapered according to clinical requirements. All patients received postoperative anticoagulant therapy, initially with heparin infusion (15–25 units/kg/h), followed by warfarin in combination to daily oral aspirin (3–5 mg/kg/day).

### Follow−up

Patients with an HM3 device received scheduled follow−up care in accordance with the institution's protocol. In the immediate postoperative period, daily monitoring of vital signs, evaluation of laboratory values, and assessment of device parameters were performed. Patients with evidence of pulmonary hypertension on postoperative echocardiography were initiated on oral Sildenafil (1–2 mg/kg/dose every 6 to 8 h) and or inhaler Iloprost (2.5 mcg/dose every 4–6 h). During the first month post−implantation, patients were followed up with weekly visits. These visits include comprehensive physical examinations, age−appropriate developmental assessments, and specific blood tests. Pediatric growth charts were used to monitor changes in height and weight. The team addressed concerns related to the child's adaptation, including psychosocial aspects. From the second to the sixth month, monthly evaluations focus on pediatric growth and development. Continuous monitoring of coagulation profiles and hematologic parameters was conducted, with adjustments to medication regimens as necessary. Pulmonary hypertension treatment was discontinued based on echocardiographic measurements during follow−up.

### Statistics

Data were analyzed using SPSS (Statistical Package for the Social Sciences) version 25 (SPSS Inc, Chicago, IL, USA). Categorical variables were expressed as numbers and percentages. Continuous variables were expressed as medians and interquartile ranges (IQR).

## Results

### Patients

We identified 11 patients (45% males) who underwent HM3 implantation during study period. At the time of surgery, the patients had a median age of 14 years (IQR 11–17), a median weight of 47 kg (IQR 28–50), a median height of 159 cm (IQR 135–165), and a median body surface area of 1.36 m^2^ (IQR 1.07–1.53). All patients were diagnosed with dilated cardiomyopathy and categorized with PEDIMACS profiles ranging from one to three. Coronary artery pathologies were evaluated using coronary CT angiography or conventional angiography in all patients. Cardiac biopsy results obtained during surgery revealed two cases of myocarditis, 1 case of focal eosinophilic myocarditis, 7 cases of myocardial hypertrophy, and 1 case of ischemic myocardial tissue. Preoperatively, 4 patients exhibited life−threatening arrhythmias. Ventricular tachycardia was detected in 2 patients, with one receiving an implantable cardioverter−defibrillator. Two patients experienced ventricular fibrillation leading to cardiac arrest, necessitating cardiopulmonary resuscitation. Table 1 provides a summary of the patients’ demographics and baseline echocardiographic parameters.

**Table 1:** Patients demographics and baseline echocardiographic parameters.

Age		Weight	Height	BSA	LVED	EF	TAPSE	Creatinin	T. Bilirubin	NT-proBNP	Ventilator	PEDIMACS	Arrhythmia
(years)		(kg)	(cm)	(m^2^)	(cm)	(%)	(mm)	(mg/dl)	(mg/dl)	(pg/ml)	Support >7 days	Profile	
Patient 1	17	50	165	1.53	6.1	15	14	0.59	0.78	7,960	–	2	–
Patient 2	17	47	162	1.48	6.7	12	24	0.67	0.84	11,649	–	2	VT
Patient 3	12	30	135	1.07	5.6	15	9	1.24	2.59	57,509	+	1	–
Patient 4	14	28	161	1.18	5.4	10	11	0.6	0.56	8,841	–	2	–
Patient 5	12	29	159	1.19	5.5	20	13	0.34	0.5	3,289	–	2	–
Patient 6	11	47	145	1.36	6.8	15	15	0.5	0.49	2,504	–	2	VF
Patient 7	9	24	130	0.93	5.5	20	8	0.75	2.1	33,377	–	1	–
Patient 8	17	60	178	1.75	7.2	15	14	0.99	0.66	3,618	–	2	–
Patient 9	14	47	159	1.46	6.7	10	13	0.61	1.04	2,980	–	2	VT
Patient 10	7	22	120	0.86	5.5	20	12	0.32	0.27	9,695	–	2	VF
Patient 11	18	61	175	1.75	7.3	20	12	0.87	0.91	4,936	–	3	–
Total Median (IQR) or *N* (%)	14 (11−17)	47 (28–60)	159 (135–165)	1.36 (1.07–164)	6.1 (5.5–6.8)	15 (12–20)	13 (11–14)	0.61 (0.5–0.87)	0.78 (0.5–1.04)	7,960 (3,289–11,649)	1 (9.1)	2 (2–2)	4 (36.7)

BSA: body surface area; LVED, left ventricular end diastolic diameter; EF, ejection fraction; TAPSE, Tricuspid Annular Plane Systolic Excursion; PEDIMACS, The Pediatric Interagency Registry for Mechanical Circulatory Support; VT, ventricular tachycardia; VF, ventricular fibrillation.

### Procedure

The median cardiopulmonary bypass time was 80 min (IQR 70–87). The patient with the lowest body weight had an open sternum post−surgery, which was closed at 48 h postoperatively. In 10 patients, the sternum was closed intraoperatively.

### Postoperative case

The median ICU stay was 14 days (IQR 6–32), with 11 patients receiving inotropic support for a median of four postoperative days (IQR 3–8). 11 (100%) patients received pulmonary hypertension treatment for a median of 45 postoperative days (IQR 30–65). Early postoperative complications included cardiac tamponade in 1 patient and pleural effusion in 2 patients, all managed surgically. Three patients developed polyuria following device implantation. These patients were consulted with the pediatric nephrology department. Their fluid balance was closely monitored to prevent electrolyte imbalances. The polyuria was interpreted as part of the recovery phase of acute kidney injury due to the improved systemic circulation post−device implantation. Over time, the polyuria resolved in these patients. In the postoperative period, 3 patients developed right ventricular failure and were treated with intravenous positive inotropic support. Right ventricular failure in two patients was attributed to their low PEDIMACS profile, while in one patient; it was linked to the genetic Carvajal syndrome affecting the right heart. Intraoperative, postoperative and follow−up data of patients implanted with HM3 are summarized in Table 2.

**Table 2:** Intraoperative, postoperative and follow−up data of patients implanted with HM3.

	Bypass duration (min)	Device average RPM	ICU stay (days)	Post-op IV inotropes (days)	HSD (days)	Early postoperative complications	PHT treatment	RHF	Liver failure	Kidney failure	Biopsy	FU duration (days)	Outcome/Death
Patient 1	70	5,200	6	3	52	Pleural effusion	Sildenafil	–	–	–	Ischemic myocardium	90	Discharged on device
Patient 2	88	4,300	10	4	10	–	Sildenafil	–	–	–	Myocyte hypertrophy	10	Heart transplantation
Patient 3	84	5,100	28	28	28	Pleural effusion	Iloprost	+	+	+	Myocyte hypertrophy	28	Death (sepsis)
Patient 4	80	5,000	33	14	69	Polyuria	Sildenafil Iloprost	+	–	–	Myocyte hypertrophy	300	Discharged on device
Patient 5	87	4,900	6	3	22	–	Iloprost	–	–	–	Myocarditis	210	Discharged on device
Patient 6	75	5,100	12	4	65	Polyuria	Sildenafil Iloprost	–	–	–	Myocyte hypertrophy	180	Discharged on device
Patient 7	70	4,600	32	4	56	Polyuria	Sildenafil Ilioprost	–	–	–	Myocarditis	180	Discharged on device
Patient 8	73	5,500	14	3	40	^a^S. aureus	Sildenafil Iloprost	–	–	–	Myocyte hypertrophy	150	Discharged on device
Patient 9	90	4,600	48	8	90	Tamponade	Sildenafil Iloprost	+	–	–	Myocyte hypertrophy	120	Discharged on device
Patient 10	85	4,600	32	3	90	–	Sildenafil Iloprost	–	–	–	Myocyte hypertrophy	120	Discharged on device
Patient 11	52	5,100	6	3	14	–	Sildenafil Iloprost	–	–	–	Myocyte hypertrophy	90	Discharged on device
Total Median (IQR) or *N* (%)	80 (70–87)	5,000 (4,600–5,100)	14 (6–32)	4 (3–8)	52 (22–69)	6 (54.5)	11 (100)	3 (27.2)	1 (9.09)	1 (9.09)	11 (100)	150 (90–210)	Death: 1 (9.1)

FU, Follow−up; HSD, hospital stay duration; PHT, pulmonary hypertension; RHF, right heart failure; RPM, revolutions per minute.

^a^ Positive postoperative blood culture.

### Follow−up

The median follow−up period of the patients was 150 days (IQR 90–210). In two patients with warfarin intolerance or suboptimal INR levels, low molecular weight heparin was used instead of warfarin. In one patient, persistent fever prompted blood cultures, revealing growth of Staphylococcus aureus. The patient received targeted antibiotic therapy for 6 weeks, with subsequent negative culture results. Another patient developed a driveline infection, which was treated with IV antibiotics after identification of MRSA.

No patients experienced pump thrombosis, stroke, seizure, neurological deficit, or pump replacement requirement. During follow−up, one patient exhibited a thrombus in the right atrium due to non−compliance with warfarin therapy, which resolved with anticoagulant therapy revision. Two patients who had arrhythmia before the procedure had unsustained VT during follow−up, and follow−up was continued with amiodarone treatment.

One patient was bridged to heart transplantation, while nine patients are currently being followed up with the HM3 and are awaiting transplantation. Unfortunately, one patient died due to postoperative sepsis. This patient had a preoperative PEDIMACS score of one and underwent surgery with concurrent kidney and liver failure.

## Discussion

VADs hold a significant place in the treatment of ESHF, with their usage frequency increasing daily (10). Ongoing improvements in device technology have allowed for wider usage, fewer complications, and better survival rates (11). The use of VADs is more prevalent in adult patients. In pediatric patients, the device size's incompatibility with the patient limits its usage. The major limitation of HM3 implantation in smaller patients is the limited space in the thoracic cavity and the potential danger of pump positioning being compromised when closing the chest (12).

Results from The Advanced Cardiac Therapies Improving Outcomes Network (ACTION) reported a series of 35 patients with congenital pathologies who underwent HM3 implantation (7). The average age was 15.7 years (range 8.8–47.3 years), the lowest patient weight was 19.1 kg, and the BSA was 0.78 m^2^. Similarly, in our study, the smallest patient weighed 22 kg with a BSA of 0.86 m^2^, and the patient was taken out of surgery with an open sternum, which was closed 48 h later. As patient size decreases, the implantation of the device becomes more challenging, and the risk of complications increases (13). The median ICU stay duration after HM3 implantation was found to be 14 days, consistent with the literature (7).

HM3 is a reliable LVAD with low rates of adverse events related to thrombosis and embolic incidents (5). In our study, no pump thrombosis, ischemia, or stroke was observed in any patient following HM3 implantation. Given that thrombosis is an expected complication in patients with dilated cardiomyopathy, aspirin and low molecular weight heparin are started in patients with an ejection fraction <25% in our institution. Despite this, intramural and atrial thrombi were observed in preoperative echocardiography. Thrombi were visualized using intraoperative transesophageal echocardiography and were cleared by an experienced surgical team. Postoperatively, patients’ anticoagulant treatments were closely monitored, and only one patient had a right atrial thrombus due to non−compliance with the warfarin diet. This patient's anticoagulant therapy was adjusted to low molecular weight heparin, leading to the thrombus regression. No pump thrombosis was observed even in the patient with atrial thrombus.

HM3 device settings were adjusted according to the patient's clinical and echocardiographic evaluations. Heart failure treatments were continued in patients after HM3 implantation. An adult patient study showed that after left VAD implantation, patients required right ventricular support device implantation or early or long−term intravenous inotropic support for right ventricular support (14). Following HM3 implantation, positive inotropic therapy was used to support the right heart in patients with right ventricular failure. During follow−up, no patient required right ventricular assist device implantation.

Pulmonary hypertension due to left heart failure is the most common etiology of pulmonary hypertension. In patients with heart failure and reduced ejection fraction, pulmonary hypertension is associated with decreased functional capacity and increased mortality (15, 16). We diagnosed elevated pulmonary artery pressures in patients with right ventricular failure through echocardiographic and clinical evaluations and initiated single or dual pulmonary hypertension treatments to reduce the resistance against the right ventricle.

Studies have shown that renal functions improve after VAD implantation (17). In our institution, where VAD implantation has been performed for a long time, it was observed that clinical recovery was faster and renal functions improved earlier following HM3 implantation compared to other devices. Patients experienced a polyuric phase in the early postoperative period. Detailed investigations revealed no pathology. It was thought that polyuria developed due to the improvement in renal functions after device implantation. Compared to other devices, this difference may be due to the intrinsic pulsatility of HM3.

In the literature, cases were generally reported from developed countries and limited centers. Our study is the first study on the use of HM3 in pediatric patients from Turkey. In Turkey, finding donors for heart transplants is challenging. The difficulty increases, especially as the patient age decreases. Only one out of 11 patients underwent a heart transplant. Nine patients continue to be monitored with the HM3. According to our experience, these patients will also be monitored with a VAD for a longer period. Preoperative PEDIMACS profiles 1–2 were shown to have increased mortality compared to higher profiles (5). In our study, the only patient who died from sepsis had a low PEDIMACS profile. Therefore, our findings might support the claims that HM3 implantation before further deterioration of the PEDIMACS profile positively affects mortality.

### Limitations

The limitations of our study include its retrospective design and the small number of patients. However, device−patient incompatibility limits its use in the pediatric patient population. Prospective studies with larger patient numbers and long−term outcome evaluations are needed.

## Conclusion

In conclusion, the HM3 device is a safe and effective treatment option for patients with ESHF. With the right timing and patient selection in the use of HM3, patients with ESHF can be saved or successfully bridged to heart transplantation.

## Data Availability

The raw data supporting the conclusions of this article will be made available by the authors, without undue reservation.
